# A narrative review on critical roles of iron levels in skin tone, aging, and photoaging

**DOI:** 10.1097/JW9.0000000000000243

**Published:** 2026-01-12

**Authors:** Xi Huang, Mariia Alavi, Lung-Chi Chen

**Affiliations:** a R & D Department, FE:I Beauty Tech, Inc., dba i-On By Dr. Xi, New York City, New York; b Division of Environmental Medicine, Department of Medicine, New York University School of Medicine, New York City, New York

**Keywords:** iron, oxidative stress, skin aging, skin photoaging, skin tone

## Abstract

**Background:**

The skin, as the body’s largest organ, reflects the passage of time through wrinkles, fine lines, uneven skin tone, and solar lentigines (commonly known as age spots). While chronological aging, defined by the inevitable progression of time, is well understood, biological aging also significantly impacts the skin.

**Objective:**

Examine and highlight iron’s historically overlooked role, as it has now emerged as a pivotal factor in biological aging, influencing skin tone, aging, and photoaging.

**Methods:**

A literature review from PubMed was conducted to address a significant gap in understanding the role of iron in skin aging, photoaging, and skin tone. We focused on key discoveries that highlight iron as a critical upstream factor influencing these processes.

**Results:**

Increasing evidence demonstrates that the buildup of excess iron in the skin is likely linked to the formation of oxidants and discoloration, leading to dull, uneven skin tones and contributing to the aging process. Iron, which remains in the skin for 60 days—twice the typical skin turnover period of 26 days—could lead to iron accumulation as we age.

**Limitation:**

More clinical trials are necessary to establish a cause–effect relationship between iron and skin tone, aging, and photoaging.

**Conclusion:**

Addressing iron-related discoloration and oxidative stress is crucial for improving skin health and appearance, providing a more comprehensive approach to skincare and treatment.

**What is known about this subject in regard to women and their families?**

- Iron is an essential transition metal crucial for normal bodily function.
- Young women, especially those menstruating, often have lower iron levels that can lead to deficiency.
- Factors like melanin production, oxidative stress, and sun exposure are known to influence skin tone, aging, and photoaging.

**What is new from this article as messages for women and their families?**

- Iron is primarily found in colorful proteins such as hemoglobin, ferritin, and hemosiderin, which accumulate under the skin and can affect skin
- tone.
- Because of the known medical condition of iron deficiency, the concept of excess iron, stored in proteins like ferritin and hemosiderin, has been historically overlooked.
- Excess iron contributes to oxidative stress by generating reactive oxygen species through reactions like Fenton and Haber-Weiss.
- Two-thirds of the body’s iron is excreted through the skin, and one-third is metabolized via the gastrointestinal tract.
- Higher levels of excess iron during menopausal transition and pregnancy could contribute to skin aging and pregnancy melasma.
- Sun exposure degrades ferritin, leading to oxidative stress and photoaging, and enhances collagenase-1 activation, initially causing wrinkles and eventually thinning the skin.
- While antioxidants neutralize oxidants, continuous iron presence can overwhelm these defenses, making protection ineffective over time.
- Traditional chelators remove free iron but cannot access ferritin- and hemosiderin-bound iron, limiting their impact on skin aging.
- Innovative technologies are needed to target ferritin and hemosiderin and stop oxidation before free iron forms.

## Background

As the largest organ of the human body, the skin vividly reflects the passage of time through wrinkles, fine lines, uneven skin tone, and solar lentigines. While chronological aging—marked by the relentless march of time—is a well-known inevitability, biological aging plays an equally crucial role. This form of aging, influenced by physiological and environmental factors, can be actively intervened. By understanding and managing these factors, the aging process can be proactively slowed and mitigated.^1^

Comprehending both aspects of aging is vital for a holistic approach to skin health. It is not just about combating wrinkles or maintaining an even skin tone; it is about preserving the skin’s overall health and vitality. This includes addressing photoaging, the damage caused by sunlight, and ensuring the skin remains protective and functional. Embracing this understanding allows us to recognize skin health as a vital aspect of overall well-being, far beyond aesthetics.

One of the pivotal factors in biological aging is iron, which can significantly influence skin tone, skin aging, and photoaging.^2,3^ Yet, its role has been largely overlooked. Most of us focus on melanin for skin tone, oxidative stress for skin aging, and sunscreens for photoaging protection. By doing so, we risk neglecting this crucial risk factor that warrants a better understanding to ensure our patients receive more comprehensive treatment.

From an aesthetic perspective, we know that skincare treatments, including color cosmetics and aesthetic procedures, often provide only superficial solutions. By prioritizing long-term skin health through clinical approaches, we can achieve not only wellness but also lasting beauty. Grasping the role of iron in skin health is essential to achieving this comprehensive goal. Here we delve into the reasons why understanding iron’s impact is so critical.

## Methods

Our investigation into the role of iron in skin aging, photoaging, and skin tone began with a comprehensive literature search across PubMed, the National Library of Medicine, the Library of Congress, SCOPUS, and the Cochrane Library. Among these databases, PubMed yielded the greatest number of results. As of August 28, 2025, a search for the keyword *“*iron*”* returned 297,872 publications, underscoring its broad significance in medicine. In comparison, searches for “skin aging,” “photoaging,” and “skin tone” yielded 26,245, 4,882, and 25,200 results, respectively. When combined with “iron,” the number of results was far more limited: 109 for skin aging, 36 for photoaging, and 228 for skin tone. By contrast, replacing “iron” with “oxidation” produced substantially more results—3,048 for skin aging, 1,202 for photoaging, and 1,027 for skin tone.

This stark difference highlights a critical gap in the current understanding of iron’s role in skin health. While oxidation is widely recognized as a key factor in aging and photoaging, it is, in fact, the downstream effect of iron-mediated Fenton and Haber-Weiss reactions. This indicates a broader oversight in the research, where the upstream role of iron is underexplored.

Given this gap, a systematic review would not adequately emphasize the nuanced understanding we seek to present. Instead, we have adopted a targeted approach, selecting pivotal discoveries over time that illustrate iron’s fundamental role in redox reactions, its excretion through the skin, its involvement in photoaging, and its contribution to collagenase-1 activity, wrinkle formation, and changes in skin tone. This selection process underscores our hypothesis that iron functions as a critical upstream factor in the oxidation processes that lead to skin tone changes, skin aging, and photoaging.

## Results

Numerous important discoveries have been made in the field of iron metabolism and its impact on skin tone, aging, and photoaging. Due to space limitations, Table 1 presents a selection of the most relevant literature, organized as a timeline. The following is a summary of findings leading to the conclusion of the critical role of iron levels in these processes.

**Table 1:** Overview of the key discovery of iron’s role in skin health and its impacts*

Year	Scientist	Discovery	Implications
1894	Fenton^4^	Iron (Fe) catalyzes hydroxyl free radical (OH·) formation	Causes oxidative damage to proteins, lipids, and DNA in cells, tissues and organs
1965	Weintraub^5^	Iron is excreted through skin exfoliation	Skin, the largest organ in the body, is susceptible to iron-mediated oxidative damage
1999	Pourzand^6^	Ultraviolet A (UVA) causes oxidation by degrading ferritin	UVA-mediated skin photoaging is caused by iron released from ferritin
2011	Huang^7^	Iron and UVA synergistically enhance collagenase-1 activity	Increased collagenase-1 activity leads to wrinkle formation and skin thinning

* The root cause of skin tone variations, aging, and photoaging might be iron. This groundbreaking discovery is the culmination of over 130 years of dedicated research.

### Iron as the best-known transition metal catalyzing oxidant formation

Transition metals are a group of metals located in the middle of the periodic table. They are called transition metals because they form a “transition” between the metals on the left side of the periodic table and the nonmetals on the right. These metals are versatile and can easily change valences. For example, iron can transition between ferrous (Fe^2+^) and ferric (Fe^3+^) ions, enabling crucial functions in producing oxidants, such as hydroxyl free radicals (OH^•^), through the Fenton reaction, which was discovered in the 1890s:^4^


$$Fe^{2+}+H_{2}O_{2}\text{---}\text{--}\to Fe^{3+}+OH^{-}+OH^{•}$$

In 1932, Fritz Haber and his student Joseph Joshua Weise further discovered that hydrogen peroxide (H_2_O_2_) and superoxide (O_2_^−•^) can generate OH^•^ through the following reaction,^8^ now known as the Haber-Weiss reaction:


$${O_{2}}^{•-}+H_{2}O_{2}\;\text{---}\text{-}\to O_{2}+OH^{-}+OH^{•\;}\left(Fe\;as\;a\;catalyst\right)$$

The reaction proceeds slowly under normal conditions. However, in the presence of iron ions, the reaction rate increases significantly, making it biologically relevant.^9^ All reactants involved in this process—H_2_O_2_, O_2_^−^, and Fe—are present in the human body. Consequently, both the Fenton reaction and the Haber-Weiss reaction are critical sources of oxidants within our biological systems.^10^ With its retention time in the skin exceeding 60 days, this highlights the importance of addressing iron in skincare.^5^

### Iron as the most abundant transition metal in the human body

Iron is the most important and the most abundant transition metal in the human body, essential for DNA synthesis, oxygen transport from the lungs to other organs, and critical in many heme-containing enzymatic activities.^11^ The human body contains approximately 4 grams of iron, significantly more than the second most abundant transition metal, copper, which averages around 80 milligrams. Thus, the iron content in our bodies is 50 times that of copper.^12^

In the body, iron can be categorized into 2 parts: essential iron, crucial for bodily functions and transport, and excess iron, primarily stored for future use but potentially damaging to beauty and health.^13,14^ This review focuses on excess iron. Alternatively, iron is distributed across 3 primary areas: essential functions in bodily processes and transport and stored for future needs.^15^ The distribution of iron throughout the body is as follows: bone marrow, 300 mg; muscle, 300 mg; splenic macrophages, 600 mg; liver, 1000 mg; and circulating RBCs, 1800 mg.^16^ About 75% of the body’s iron is functional, primarily found in hemoglobin, myoglobin, and heme-containing enzymes.^15,16^ Hemoglobin, which carries oxygen, contains 3.3 mg of iron per gram, while myoglobin, present in all skeletal muscle and heart cells, contains a smaller amount.^17^ Additionally, heme-containing enzymes, such as cytochromes and ribonucleotide reductase, hold a minor fraction (6-8 mg) of iron. Most cycling iron comes from the spleen, where RBCs are destroyed by splenic macrophages; the iron is recycled to the bone marrow via transferrin. Functional iron is crucial for the metabolism of all cells and is indispensable for life.

Approximately 0.1% of the body’s iron is involved in transport, primarily as Fe^3+^ bound to transferrin in plasma.^17^ Transferrin binds Fe^3+^ in a physiological form, preventing “free” iron, which is oxidatively active and toxic, and facilitating its delivery to cells *via* transferrin receptors (TfR). TfR1 is expressed in all cell types, while TfR-2 is mainly found in liver cells.^17^ Both functional and transport iron are essential for the body’s physiological processes and are labeled as essential iron in this article to distinguish it from excess iron in ferritin and hemosiderin.

About 25% of the body’s iron is stored, mainly in the form of ferritin, with a single ferritin molecule capable of holding up to 4,500 iron atoms.^18^ Typically, 60% of ferritin is believed to be stored in the liver and 40% in the reticuloendothelial system, classifying this iron as excess iron. However, the accumulation of excess iron in the skin has been largely overlooked, despite its role as the body’s largest organ and a key site of iron excretion and storage. This important aspect of skin iron storage and its implications for aging will be discussed in the following section. Hemosiderin, an iron-protein complex derived from ferritin and hemoglobin, can form semi-crystalline aggregates, especially in the liver, spleen, bone marrow, brain, and potentially the skin.^19^ In cases of iron overload, hemosiderin levels can increase up to tenfold compared to ferritin. The iron in hemosiderin is more challenging to mobilize than the iron in ferritin.^20^

### Skin as the major body organ for iron excretion

In human physiology, iron homeostasis is regulated through 2 key axes: the hepcidin–ferroportin (Fpn) axis at the systemic level and the transferrin-TfR1 axis at the cellular level.^21–23^

At the systemic level, hepcidin, a 26-amino acid peptide hormone, acts as a negative regulator of iron absorption and distribution.^24^ When iron levels are elevated, hepcidin production increases. Hepcidin binds to Fpn, the only known cellular iron exporter, leading to degradation of Fpn.^25^ This prevents iron absorption from the diet in the intestine and blocks the release of iron from tissue stores into the bloodstream.

At the cellular level, iron transport is regulated by the binding of transferrin, the major iron-carrying protein in the blood, to TfR1 on cell surfaces.^15,21^ This interaction facilitates the uptake of iron from the extracellular environment into cells, ensuring sufficient iron availability for cellular functions.

These 2 regulatory systems work together to maintain iron balance in the body. Importantly, this mechanism also influences iron excretion through the skin, which we will discuss later.

The human body typically loses approximately 1-2 mg of iron daily. Early studies suggested that the gastrointestinal tract was not the primary organ for iron excretion, but this was not proven in the 1930s due to the insensitivity of iron assays.^26^ Using whole-body counting techniques in the 1960s to monitor radioactive Fe^59^ following intravenous injection, it was discovered that one-third of body iron is excreted via the intestines, while two-thirds is eliminated through the skin.^5^

Interestingly, in a clinical trial with iron-deficient or anemic conditions, there is no significant difference in iron excretion in the skin, including the duration of iron retention, between individuals with iron deficiency or anemia and healthy subjects with normal iron levels.^27,28^ This observation suggests that individuals with anemia or lower systemic iron levels may not be more susceptible to slower skin aging due to iron-mediated oxidative stress. However, this possibility requires further investigation to be fully understood.

On average, iron remains in the skin for 60 days, which is twice the duration of the skin’s natural turnover rate of 26 days.^5,28^ In contrast, the retention of radioactive iodine (I^125^) in the skin is less than 24 hours, as its primary target is the thyroid gland.^5^ The longer retention time of iron in the skin suggests that iron may have a substantial impact on skin health and function.

Following the intradermal injection of Fe^59^, biopsy specimens were taken from the forearms. It was found that the external loss of iron was demonstrated during this period by significant radioactivity in heat-stimulated sweat collections and keratin stripping from the forearm using Scotch tape. In addition, excision of the injection site on the 14th day resulted in an average of 31% drop in arm radioactivity.^5^ Radioautographs of the skin sections showed selective localization of iron in the epithelial cells of the epidermis, eccrine sweat glands, sebaceous glands, and hair follicles.^5^ There was no significant radioactivity in the connective tissue, likely the site of the needle tip during injection.

The highest radioactivity in the epidermis was found in the basal layer of the stratum malpighii, decreasing toward the stratum granulosum.^5^ This gradient is due to the higher cell concentration in the basal layer. In hair follicles, Fe^59^ was primarily localized in the outer root sheath. The secretory tubules of the eccrine glands showed more radioactivity than the ductal epithelium. In the sebaceous glands, the highest activity was observed at the gland periphery. These findings underscore the skin’s critical role not only in maintaining iron homeostasis but also as a principal site of iron deposition, which may contribute to skin tone, skin aging, and photoaging. Here are some potential reasons.

### Iron proteins as contributors to skin tone and discoloration

Iron proteins play a significant role in skin tone and discoloration due to their inherent colors. Ferritin, which stores excess iron, appears brown. Excretion of iron by the skin is in the form of ferritin. Hemoglobin, an oxygen-transporting protein in red blood cells, gives blood its red color because of iron and oxygen.^13,14^ Purpura (bruises) are result from blood pooling under the skin due to vessel damage, leading to hemoglobin accumulation and visible discoloration. Sleep deprivation and Ultraviolet exposure can exacerbate dark circles under the eyes, often partly due to hemoglobin deposits from leaking micro blood vessels.^29^ Additionally, solar lentigines, also known as age spots, liver spots, or sunspots, are named after their similar color to the liver color. Hemosiderin—a complex of hemoglobin and ferritin—is high in liver and kidney and can contribute to various skin pigmentations from tan to dark brown.^30^ Physiologically unrelated to liver, solar lentigines are caused by sun damage over time and are most common in people over 55 with significant cumulative sun exposure. Leaking hemoglobin and excretion of ferritin at the sunburn site are likely to form hemosiderin and are worth further studying.^31–34^

Chemical peeling of the face using acids such as tranexamic acid, glycolic acid, lactic acid, azelaic acid, and trichloroacetic acid is widely used to treat solar lentigines and melasma.^35,36^ Besides providing mild exfoliation, these acids may also dissolve iron from iron proteins, contributing to their effectiveness. Considering the role of iron proteins in solar lentigines opens additional treatment options.

Heme oxygenase-1 (HO-1) inducers, such as quercetin, curcumin, carnosic acid, fucus seaweed extract, resveratrol, epigallocatechin gallate, and caffeic acid phenethyl ester, have been found to induce HO-1.^37^ The beneficial effects of HO-1 induction are likely associated with the degradation of the heme group in hemoglobin and hemosiderin, and the production of anti-inflammatory products like biliverdin and carbon monoxide (CO).^38,39^ These HO-1 inducers can potentially help reduce dark circles under the eyes and lighten solar lentigines.

Therefore, besides melanin, iron proteins are important contributors to skin tone and discoloration. Understanding their impact can lead to more effective treatments for improving not only skin appearance but also overall skin health. Reducing iron in the skin can lead to less pigmentation and healthier skin.

### Iron as a critical contributor to photoaging

Research has shown that ferritin can undergo immediate degradation in human FEK4 fibroblasts when exposed to daily doses of 100, 250, and 500 kJ/m^2^ UVA radiation.^6^ This exposure releases significant amounts of “free” iron, which then facilitates the formation of harmful oxidants. These oxidants contribute to oxidative stress, which is a key factor in skin photoaging.^40,41^ It was previously understood that photoaging is caused by UVA-mediated oxidant formation. This study points out that ferritin is the intermediate between UVA and subsequent oxidation, which leads to photoaging.^6^ Because of this finding, removing iron from the skin, in addition to antioxidants and sunscreen, will probably provide a more potent solution in preventing photoaging.

Furthermore, the interaction between ferritin and UVA radiation also increases the production of matrix metalloproteinase-1 (MMP-1) in primary human dermal fibroblasts.^7^ MMP-1, also known as collagenase-1, is an enzyme directly linked to the breakdown of collagen and other extracellular matrix components in the skin.^42^ This enzymatic activity exacerbates skin photoaging by digesting collagen content, promoting wrinkle formation over the short term and skin thinning over the long term. Even more interestingly, it was found that normal human epidermal keratinocytes can release tumor necrosis factor-alpha into the media, which further enhances MMP-1 in fibroblasts.^7^

These findings reveal a clearer mechanism: ferritin serves as a critical intermediary between UVA exposure and the enzymatic destruction of collagen. Therefore, targeting iron removal in the skin could interrupt this harmful cycle at its source. With this deeper understanding, skincare solutions that combine iron removal technology, antioxidants, and sunscreen may offer a more comprehensive defense. Such approaches could allow individuals to enjoy the benefits of sunlight—such as improved mood, enhanced vitamin D synthesis, and stronger bones—without suffering the harmful effects of photoaging and sunburn caused by UVA and UVB exposure.^43,44^

### Additional evidence of iron’s critical role in skin tone, aging, and photoaging

During the menopausal transition, women’s skin often becomes thin, dry, and wrinkled due to changes in collagen and elastin content and a decreased ability to retain fluids.^45,46^ While estrogen deficiency has long been considered the primary factor in menopausal skin aging,^47,48^ estrogen replacement therapy, either through topical or oral application, does not show significant effects on skin wrinkle reduction and collagen synthesis.^49–51^ There is another significant change occurring during this period: a substantial increase in iron levels.^45^ Although estrogen levels decrease by about 90%, serum ferritin levels increase by 100 to 200% from before menopause to after menopause. More interestingly, healthy human skin biopsy samples have shown that ferritin levels are 42% higher in postmenopausal skin, while vascular endothelial growth factor and total antioxidant capacity are 45% and 34% lower, respectively, compared with premenopausal women.^52^

These findings point to a broader need to reevaluate the role of iron in menopausal skin aging. While estrogen decline has been the primary focus, the substantial rise in ferritin levels during menopause introduces a secondary, yet critical, factor in skin aging. Excess ferritin-bound iron can catalyze the formation of reactive oxygen species through redox cycling, exacerbating oxidative damage in skin tissues. This oxidative stress, combined with reduced antioxidant capacity and impaired vascular support, may contribute to the thinning, dryness, and delayed wound healing observed in menopausal skin.^52^ Although systemic iron accumulation and oxidative stress have been well documented in postmenopausal women,^53^ the skin’s role as both a storage site and an excretory organ for iron has been largely overlooked. These insights suggest that targeting excess iron accumulation in the skin could complement hormone therapies and provide a novel approach to preserving skin health during menopause. Further research is needed to fully understand the interplay between iron homeostasis and skin aging in this population.

Melasma can occur during pregnancy and is often attributed to an increase in melanin production due to hormonal changes. However, pregnancy is also characterized by the sudden cessation of menstruation, during which a woman typically loses about 60 mL of blood (equivalent to ~30 mg of iron) each month.^54,55^ Over the course of a 10-month pregnancy, this pause in blood loss may result in an estimated iron retention of up to 300 mg. This rapid increase in iron over a relatively short period, compared with the gradual iron changes during the menopausal transition, which can last up to 10 years, may have a significant impact on the skin. Additionally, if iron supplements are taken during pregnancy, their influence on pregnancy-related melasma could be even greater and warrants further investigation.

Skin changes can be a symptom of hemochromatosis, a condition characterized by iron overload in the body.^56^ These changes may include a gray-to-brown discoloration of the skin, particularly in sun-exposed areas such as the face, hands, and forearms. Additionally, the skin may become dry and itchy to varying degrees. This phenomenon provides further evidence of the role of iron in skin health. Hemochromatosis represents a severe case of iron overload, making it an extreme example of how excess iron can affect the skin.

### Molecular mechanisms of iron excretion from the skin

Cellular iron homeostasis is controlled by 2 primary mechanisms: the transferrin and TfR axis, which regulates iron inflow, and the hepcidin and Fpn axis, which regulates iron outflow.^11,22^

To investigate the roles of TfR, hemochromatosis Fe, and skin turnover in regulating iron content in the epidermis, researchers used transgenic mice with stratum-specific expression of the human TfR.^57,58^ They found that epidermal iron content increased with age and was 2-3 times greater in 8-week-old transgenic mice compared with littermate controls.^57,58^ Ferritin expression, which was low in normal epidermis, was significantly increased in the transgenic mice, indicating it as the likely storage site for excess iron. These findings suggest that higher expression of TfR and higher skin turnover rate, but not hemochromatosis Fe gene expression, are crucial for iron excretion from the body to skin.

In another study, researchers used mice carrying the K5-Cre transgene and Fpn1-floxed alleles (Fpn^Epi-KO^) to examine the role of ferroportin in iron outflow.^59^ They discovered that the iron content in the epidermis and squames was significantly higher in Fpn^Epi-KO^ mice than in control mice. Additionally, Fpn^Epi-KO^ mice showed a more rapid decrease in blood hemoglobin concentration than control mice when on a low-iron diet. These results demonstrated that the epidermis has mechanisms to excrete intracellular iron from differentiated keratinocytes into the extracellular space before reaching the stratum corneum. These studies collectively highlight the importance of both TfR and Fpn in maintaining iron homeostasis in the skin, ensuring proper regulation of iron inflow and outflow to prevent iron overload and associated skin conditions.

## Limitations and perspectives

The studies cited here strongly suggest a significant role for iron in influencing skin tone, aging, and photoaging. However, most of these studies do not establish a direct cause-and-effect relationship. A recent finding demonstrated that blood donation (phlebotomy), which reduces iron stores, can alleviate signs of skin aging by decreasing the expression of inflammatory genes and enhancing collagen resynthesis in older mice.^60^ While promising, this study was conducted in mice, and more human studies are needed to confirm that reducing iron can decrease skin iron levels, improve skin appearance, and, most importantly, enhance skin health by making it less susceptible to environmental factors such as pollution and sunlight.

Until now, antioxidants, such as vitamin C and quercetin, have been the primary defense against oxidants, attempting to neutralize them after they form.^61–63^ However, since iron is constantly present, this reaction continuously produces OH^•^, necessitating a constant supply of antioxidants. This reliance on antioxidants has 2 major limitations: (1) it lowers the total antioxidant capacity of the skin, as observed in postmenopausal skin,^52^ and (2) it requires the continuous presence of antioxidants to counteract the oxidants. Over the long term, this approach is ineffective because antioxidants are not stable and they cannot penetrate deeply into the skin.^64,65^ Although antioxidants can be taken orally, they are eventually consumed.

Chelation therapy helps bind and remove free iron, known as nontransferrin-bound iron,^66,67^ and has shown potential in treating certain skin conditions. For example, applying deferoxamine through the skin may help prevent ulcer formation and accelerate the healing of diabetic wounds, offering a promising treatment approach that could be used clinically.^68,69^ However, traditional iron chelators cannot effectively remove iron stored in ferritin, as ferritin binds iron much more tightly. As a result, these chelators may have limited effectiveness in preventing skin aging.

Both antioxidant and chelation therapies are reactive approaches, working only after oxidants are formed or iron is released and free. They help manage symptoms but do not address this underlying cause of skin aging—excess iron accumulation in the skin. To truly slow skin aging, it is essential to remove this excess iron before it fuels oxidative stress and tissue damage. What is needed is innovative technology to reduce excess iron in ferritin before it converts to skin-damaging free radicals. This proactive approach would eliminate the source of oxidant formation, leading to more effective prevention and treatment for even skin tone and reduced aging and photoaging. By addressing the root cause, we can achieve a more substantial and lasting improvement in both skin appearance and health.

This review did not address the role of iron in hair and scalp physiology. Iron is frequently cited as a key factor in hair health, and ferritin is often monitored as a biomarker, with both deficiency and excess having potential implications.^70^ However, a detailed examination of iron’s effects on hair growth and scalp conditions lies beyond the scope of this narrative review and warrants a dedicated review in the future.

## Conclusions

As we age, our complexion often becomes reduced radiance, increased sallowness, diminished translucency, with uneven discoloration. This phenomenon is likely linked to the buildup of iron in the skin. Iron’s role is paradoxical: it is essential for building strong structures yet also causes deterioration through oxidation. In the skin, oxidative damage arises from the accumulation of iron, which remains in the skin for 60 days—twice the typical skin turnover time. This buildup, coupled with a slowing exfoliation process, could significantly contribute to skin tone, aging, and photoaging. Understanding and addressing this iron-related discoloration and oxidative stress is crucial for improving skin health and appearance. However, despite emerging evidence, there is still a paucity of well-designed human studies in this area. Future clinical investigations are needed to validate these mechanisms, quantify their impact on aging skin, and evaluate potential therapeutic interventions.

## Conflicts of interest

The authors made the following disclosures: Dr. X.H. is the founder and president of FE:I Beauty Tech, Inc., dba i-On By Dr. Xi Skincare.

## Funding

None.

## Study approval

N/A.

## Author contributions

XH, MA, and LCC: Participated in research design, writing of the manuscript, and approval of the version of the manuscript to be published. XH conceived the research and participated in the writing of the manuscript and the approval of the version of the manuscript to be published. MA and LCC participated in the research design and approval of the version of the manuscript to be published.
